# Exploring Human-Tech Hybridity at the Intersection of Extended Cognition and Distributed Agency: A Focus on Self-Tracking Devices

**DOI:** 10.3389/fpsyg.2018.01432

**Published:** 2018-08-13

**Authors:** Rikke Duus, Mike Cooray, Nadine C. Page

**Affiliations:** ^1^School of Management, University College London, London, United Kingdom; ^2^Hult International Business School, London, United Kingdom

**Keywords:** hybridity, extended mind, agential intra-action, wearable activity trackers, agency

## Abstract

In an increasingly technology-textured environment, smart, intelligent and responsive technology has moved onto the body of many individuals. Mobile phones, smart watches, and wearable activity trackers (WATs) are just some of the technologies that are guiding, nudging, monitoring, and reminding individuals in their day-to-day lives. These devices are designed to enhance and support their human users, however, there is a lack of attention to the unintended consequences, the technology non-neutrality and the darker sides of becoming human-tech hybrids. Using the extended mind theory (EMT) and agential intra-action, we aimed at exploring how human-tech hybrids gain collective skills and how these are put to use; how agency is expressed and how this affects the interactions; and what the darker sides are of being a human-tech hybrid. Using a qualitative method, we analyzed the experiences of using a WAT, with a specific focus on how the tracker and the individual solve tasks, share competences, develop new skills, and negotiate for agency and autonomy. We contributed with new insight on human-tech hybridity and presented a concept referred to as the agency pendulum, reflecting the dynamism of agency. Finally, we demonstrated how the EMT and agential intra-action as a combined theoretical lens can be used to explore human-tech hybridity.

## Introduction

Throughout time, humans have utilized the capabilities and skills derived from interacting with external tools, entities, devices, and artifacts to complement their own cognitive abilities (Thacker, 2003; Dinerstein, 2006; Herbrechter, 2012; Heersmink, 2017). There are many ways that human cognition can be enhanced with external artifacts, some of which are rather mundane, including shopping-lists, books, diaries, recipes, calculators, spreadsheets, and, more recently, mobile phones. The extended mind theory (EMT) is a helpful theoretical apparatus when considering how and in which ways, cognitive processes can become extended across multiple human and nonhuman entities. The EMT reflects Clark and Chalmers (1998) argument that cognitive processes (e.g., memory, information retrieval, and processing) can take place outside of the human mind. Hence, cognitive abilities are a collection, an ensemble (Clark, 2015), of human and other external entities that together perform tasks and solve problems.

The EMT has previously been used as a theoretical lens to explore a number of different contexts, such as the musically extended mind (Krueger, 2014), spirituality and Christian life (Brown and Strawn, 2017), treatment of sex offenders (Ward, 2009), social ant behavior (Bosse et al., 2005), as well as other studies that explore cognitive integration and the extended mind (Menary, 2010), and sense-making (Thompson and Stapleton, 2009). However, studies like these that adopt the EMT are typically conceptual and do not seem to engage directly with research subjects to understand, in practice, how cognitive capabilities become distributed and contribute to the formation of hybrids. In the context of human-tech hybridity, there is a need for further research into the ways in which agency is acquired, expressed and lost as well as the darker sides of these hybrid formations.

We adopt the empirical context of people who use or have recently used wearable activity trackers (WATs) to manage their health and well-being and the trackers that collect, store, and reproduce the health and well-being data. We rely on their accounts of interacting with the WATs and are interested in their lived experiences. WATs are interesting to study in this context, as the technology has gained an on-body position, constantly capturing movements and activities with the potential to influence the person’s behaviors, decision-making, and information access. The WATs undertake certain activities with few instructions from the user, e.g., automatically starts monitoring sleep and determines which kind of physical activity the person is performing, while in other situations the person inputs information (e.g., food items), which the WAT transforms into visualizations. We seek to contribute to other recent studies (e.g., Bode and Kristensen, 2016; Etkin, 2016; Fotopoulou and O’Riordan, 2016; Nelson et al., 2016; Rapp and Cena, 2016; Smith and Vonthethoff, 2016) that have used self-monitoring and self-tracking as the empirical context to investigate various areas of human-tech engagement.

We apply the EMT and Barad’s (2003) concept of agential intra-action as a combined theoretical lens to explore how cognition is extended to the WATs to solve tasks and provide new insight, while also expressing agency (Clowes, 2018). In this way, the paper builds on the EMT by examining the non-neutrality of the WATs and how they acquire agency in particular situations, which is further conceptualized through our concept of the agency pendulum. As such, this research believes that technology can act as cognitive extensions and at the same time express agency. Hence, we put forward that distributed cognition and distributed agency can be detected when exploring human-tech interactions and that these are important to understand what it means to be a human-tech hybrid.

We posit that hybridity is not a stable condition with predetermined roles and affects. Rather, it is an ongoing process that interweaves the human biological and cognitive with the abilities of other entities. Clark (2007, p. 279) underlines how the human emerges as a “soft self,” ready to adapt and be adapted by technological others:

The realization that we are soft selves, wide open to new forms of hybrid cognitive and physical being, should serve to remind us to choose our bio-technological unions very carefully, for in so doing we are choosing who and what we are.

Using the EMT and agential intra-action, we aimed at exploring how human-tech hybrids gain collective skills and how these are put to use; how agency is expressed and how this affects the interactions; and what the darker sides are of becoming a human-tech hybrid.

The literature review starts with a focus on the EMT to explain the core concepts of extended cognition, collective problem solving, coupled systems, and non-neutrality. This is followed by a review of Barad’s (2003) agential intra-action concept.

## Literature Review

### Extended Mind

Situated approaches to understanding cognition, such as embodied, enactive, embedded, and extended, have come to challenge the traditional cognitivist paradigm (Heersmink, 2017). Situated cognition is a form of cognitive extension that can be expressed in a multitude of ways through engagement with a person’s external environment. The situated cognition movement has developed primarily since the late 1970s and offers an alternative paradigm for exploring and conceptualizing the mind (Wilson and Clark, 2009). At its core, situated approaches consider human thought as *affected* by the external socio-technological environment (Hutchins, 2014). Hence, when the external environment changes, the individual’s cognitive abilities are also impacted. Therefore, when taking a situated approach to cognition, the external socio-technological environment is an important source of influence on human thought.

Clark and Chalmers (1998) concept of extended mind is an attempt to question the locus of cognition as belonging intrinsically and only to the human mind and body. Instead, they advocate that the human mind and cognition is extended across larger systems of different kinds of entities. The focus of the extended mind is how internal and external resources operate together in “driving more-or-less intelligent thought and action” (Sutton et al., 2010, p. 525). Clark (2001, p. 134) explains:

We – more than any other creature on the planet – deploy non-biological elements (instruments, media, notations) to *complement* our basic biological modes of processing, creating extended cognitive systems whose computational and problem-solving profiles are quite different from those of the naked brain.

It is collective problem-solving, involving both a person’s internal resources, e.g., the ability to recall items to purchase from the supermarket, combined with the resources afforded by an external entity, e.g., a shopping list with items to purchase. Another example is the internal ability a person may have to find their way around the streets of London to reach a particular destination. This ability is based on the person’s prior knowledge and experience of the network of streets, shortcuts and traffic patterns. The task of getting from A to B, however, is often complemented by the directions and visualizations offered by an external resource; a digital map on the person’s smartphone, for example. Hence, to complement the human mind’s limited capacity (Norman, 1993), artifacts are created and used as scaffolding to help the person perform certain tasks. Artifacts that offer cognitive scaffolding thereby complement the human information processing capacity by providing information, resources, or capabilities, as and when required, in order to perform the task (Clark, 2015). The human-artifact hybrids gain new capacities (Wilson and Clark, 2009), which in turn affect behaviors, decision-making, and identity formation. Rowlands (2009) also believes that humans use the world around them to extract relevant information which is used to support basic functions such as perception, memory and reasons. These cognitive processes, he believes, take on a hybrid form as they “straddle both internal and external operations” (Rowlands, 2009, p. 2). This need to externalize thought in order to enhance processing capabilities through the use of complementary technologies is an inherent feature of the human experience.

Clark (2015) explains that devices, such as, for example, laptops and smartphones, can be considered bio-external devices that offer resources (e.g., information) for specific tasks, depending on the context and the level of uncertainty. Hence, the context that the human-artifact hybrid is in has an impact on the types of resources needed and the ability of each agent (internal and external) to provide and share the necessary resources. The person and the scaffolding can be so strongly coupled that they become one single cognitive system. Heersmink (2017) explains that the more a person depends on the external information to perform tasks that require cognitive abilities, the deeper the external information, or artifact that provides the information, is integrated with their internal cognitive system. In this way, it is a dynamic relationship and the extent to which the person and the artifact become a single cognitive system, is contingent on factors such as the intensity of information flow, accessibility of the scaffolding artifact, durability of coupling, amount of trust in the scaffold’s information, among other (Heersmink, 2015).

To sum up, the concept of extended mind argues for an approach to cognition that is distributed and extended across human and other entities. The surrounding environment is seen as always affecting human thought, memory, decision-making, and actions. Hence, individuals undertake tasks in collaboration with artifacts and can even become a single cognitive system. As such, the EMT considers objects, people, systems, and other external components as part of a larger cognitive system.

In the Section “Coupled Systems,” we explore the nature of the human-artifact coupling in further detail through Clark and Chalmers (1998) concept of coupled systems.

#### Coupled Systems

The concept of coupled systems is the linking of the human organism with an external entity in a two-way interaction, which creates a new cognitive system (Clark and Chalmers, 1998). These human and external entities interact in one system where each plays an active role and acquires collective behavioral competences. If external parts are de-coupled from the system, the collective competences are reduced or even lost. The external parts, or features, that are embedded in the coupled system have the ability to act and influence the overall system. Clark and Chalmers (1998, p. 51) argue that the external features possess an “ineliminable role,” as, if changed, the behavior of the person is likely to change too, even if the internal structure (e.g., the capacity to recall information, plan behavior, etc.) remains the same.

Coupling is one of the more contentious areas of the EMT and is also referred to as the coupling-constitution fallacy (Rowlands, 2009). According to Aizawa (2010), Clark’s (2008) argument that a causal dependency between a cognitive process (A) and some other process (B) can make B or A-B constitute a cognitive process is flawed. Clark (2008) has defended this core pillar of EMT by arguing that all couplings are not automatically considered to constitute an extended cognitive process. Rather, focus should be on the *effect* of the coupling and its ability to surface information that is useful within a specific situation of problem-solving. This perspective is further emphasized in Clark and Chalmers (1998) concept of active externalism. Active externalism is grounded in the belief that the external features (e.g., a book, watch, to-do-list, fitness tracker) directly impact the person and the person’s behaviors. In this way, the external features play an active role in the creation of the here-and-now and the capabilities of the human-artifact hybrid (Clark and Chalmers, 1998). Often, external features will be taken in use in order to enhance cognitive hybridization by acquiring the ability to process large amounts of information, faster and with a greater level of accuracy (Heersmink, 2017).

There are certain criteria that affect the strength of the human-artifact coupling (Clark and Chalmers, 1998; Heersmink, 2015) and therefore also the extent to which the external features become constitutive of a cognitive process. The coupling needs to be reliable, which means that the external feature or resource needs to be accessible as and when it is required. For example, a shopping list needs to be available when the person needs it in order to solve the particular task, for it to become part of the cognitive resources that the person has available to draw on (Clark and Chalmers, 1998). To create this coupling, it requires a high level of portability, and more importantly, accessibility, to ensure that the coupling is reliable. Clark and Chalmers (1998) argue that occasional decoupling, damage, loss, or malfunction does not put into question this unified cognitive system. They point to how a person’s internal cognitive capabilities may also be challenged at times (e.g., from a lack of sleep, illness, or intoxication) and as along as the external features are available when required, then that constitutes a coupling. It is further important that the information that flows from the external source is trusted by the person receiving it. If the information is not trusted, its role as a guide for action will be challenged. Heersmink (2015) adds further detail to the notion of trust. He argues that (dis)trust can be either explicit or implicit. The main difference between explicit and implicit trust is that for explicit trust, the information is consciously evaluated before determining whether it is trustworthy or not. For implicit trust, the information is assumed either trustworthy or not trustworthy. We tend to trust information implicitly if we have endorsed it in the past, if many people rely on this information to guide their action, or if it is relevant to achieving set goals (Arango-Muñoz, 2013). Tripathi (2010) explains that the more we depend on technologies to carry out or mediate our everyday activities, the more we will need to trust them to do so.

To sum up, a person and an external entity can become a new cognitive system through two-way interaction. However, the external entity needs to possess a high degree of trust, reliance, and accessibility and it must have been endorsed by the person at some point in the past in order to become part of this new system (Clark and Chalmers, 1998).

As we continue to explore facets of EMT as a perspective to understand human-WAT hybridity, it is of great relevance to consider the role the technology plays in shaping intentions and effects. We provide a brief review of the literature on the non-neutrality of technology, which leads us to further explore the agentic expressions of technological entities.

#### Non-neutrality

The concept of non-neutrality should be seen in the context of human-tech hybridization and as a contrasting perspective to the views that technology is always enhancing (i.e., positive) or that hybridization is neutral (i.e., means to an end). Verbeek (2006) explains that technologies always mediate and shape action. Ihde (1990) and Heersmink (2017) support this argument that human-tech hybridization is not neutral, as technologies do shape intentions and effects. Ihde (2004, p. 120) explains that “To take instruments either for granted or as simply transparent, is to make an implicit assumption that instruments are ‘neutral’.” Heersmink (2017) identifies three ways to understand the non-neutrality of technology. First, technologies can embody moral and political values. For example, a non-smoking sign encourages smokers not to smoke and instead adopt a behavior that is typically supported by health authorities. Second, technologies can mediate and transform experiences and perspectives on the world. The experience of being-in-the-world is affected by the technologies an individual interacts with, whether that is a motorbike, a heart monitor, or a computer game. The impact a technology has can be detected by paying attention to which aspects of an experience that it amplifies and which it reduces (Tripathi, 2010). For example, the use of whiteboards to mindmap ideas and thoughts amplifies the ability to organize, inter-relate, and prioritize information and, potentially, share it with others. Third, technologies can be seen as having unintended consequences, which are difficult to affect or change. For example, social networking platforms were designed to bring people together, but, unintentionally, have also contributed to issues of social anxiety and isolation for some users. Other unintended consequences can be seen with something as mundane as word processing software and the in-built spell checking feature. Users of this software, may experience their spelling deteriorate due to their misspelling automatically being corrected, sometimes so quickly that the person may not even notice the correction being made.

As technology becomes more ubiquitous and present in day to day tasks and interactions, there is a risk of over-reliance on the external information that the technologies provide (Carr, 2011). This may lead to a reduction in the cognitive abilities, as tasks, information storing, and problem-solving are outsourced to technologies, and therefore not learned or practiced by the individual.

The non-neutrality of technology underlines the idea that technological entities shape intentions and effects, some of which can be unintended consequences or side effects, which are unexpected. In the final section of this review of the literature, we continue to explore the role of technology in the making of human-tech hybridity.

### Agential Intra-action

Like the EMT acts to challenge the traditional cognitivist paradigm, Barad’s (2003) work on agential intra-action also takes an oppositional stance. It encourages a re-think and re-view of our relations with the external world by de-centering the dominant human actor and instead focusing on the intertwined nature of humans and other entities (Pickering, 2013).

Agential intra-action considers agency to be emergent and distributed over human and nonhuman forms. Hence, from this perspective, agency is not deterministic, absolute or, indeed, only a human practice. As such, agential intra-action also challenges the dominant human subject (Puig de la Bellacasa, 2009). Nonhuman entities, such as technology, are also seen to express agency, affect, and influence relationships as well as the collective practices, capabilities, and cognitive abilities of human-tech hybrids.

Barad’s (2003) focus is on understanding the co-created and co-constructed behaviors, decisions, and experiences, which take place within these hybrid relationships. She explains that “Agency is not an attribute but the ongoing reconfigurings of the world” (Barad, 2003, p. 818). In other words, entities, whether human or other, are seen to express agency (e.g., the ability to influence a situation) as they interact with other entities in the world. This is what Barad (2003, p. 817) refers to as the “ebb and flow of agency.” Hence, situations, relations, and identities are ongoing and evolve between actors, who through those intra-actions become and act. To understand how different human and nonhuman entities *become*, it is important to account for both human and nonhuman forms of agency (Barad, 2003). It is also important to emphasize that, in this view, agentic expressions by nonhuman entities are not purely extensions or transferals of human agency. Pickering (2013, p. 25) explains that it is useful *not* to think about agency in terms of “will, intention, calculation, and representation,” but rather in terms of performance, doings, actions, impact, and influence. In other words, agency is expressed in places of action, of consequence, of impact – in places where change occurs. Pickering (1995, p. 102) refers to this as the “open-ended dance of agency.” Hence, that it is through intra-actions that agency is enacted and that agentic expressions flow back and forth between actors. As such, human-tech (and other) relationships are always dynamic, always unfolding.

In summary, agential intra-action provides an opportunity to acknowledge nonhuman agentic expressions, doings and actions as the human and technology interact. Adopting this lens in combination with the EMT enables an investigation of how agency becomes distributed across the users and the WATs in situations of extended cognition.

## Materials and Methods

A qualitative approach to inquiry was adopted to collect and critically evaluate a multitude of perspectives from individuals who have a range of experiences with WATs. The aim of the study was not to provide generalizations across all users of WATs, but rather to explore in-depth the subjective, lived experiences, and human-WAT relationships, which each participant in this study is involved in co-creating. We intend to empirically identify and evaluate a range of experiences, actions, emotive responses, and skills attainment, which can help to illuminate what it is like to be a human-WAT hybrid. This insight, consequently, demonstrates the usefulness of adopting the EMT and agential intra-action as a set of combined theoretical perspectives and drivers to investigate people’s relationships with technology. This is a timely inquiry due to technology’s fast advancement, interactive nature, and ubiquitous involvement in a multitude of daily life experiences and decisions.

### Research Participants and Sampling

The purpose of the empirical data collection and analysis was to understand how human-tracker hybrids gain collective skills and how these are put to use; how agency is expressed and how it affects the interaction; and whether these human-tracker relationships have darker sides. In order to capture insight from users of WATs in relation to these research areas, the research team used stratified purposive sampling (Ritchie et al., 2003) to select eight female participants, living and working in the United Kingdom.

We adopted this sampling approach to ensure that our participants met specific criteria and would be able to contribute with new insight in relation to the main objectives of the study, while also being individually comparable (Bryman and Bell, 2011). This study is part of a larger research inquiry currently focused on women’s experiences of using digital devices, such as WATs, to manage health and well-being. Therefore, only female users of WATs were considered for this study, although in future studies this is likely to extend to also include male users.

Beyond gender, prior experience of using and interacting with a WAT to manage own health and well-being was a primary factor for sample selection. All participants had used a WAT for at least 6 months. In line with the stratified purposive sampling approach, we wanted to ensure some diversity of the sample and to take an inclusive approach (Ritchie et al., 2003). Hence, we further sampled according to specific usage levels. To capture a wider range of experiences, it was important not only to include individuals who were highly engaged with their WAT, but also those who had a lower engagement level and those who had become non-users after having used a WAT in the recent past. In terms of defining engagement, we identified this as a combination of two behavioral attributes. First, the extent of interest in monitoring day-to-day activities (e.g., the frequency of checking performance analytics), and second, the level of intensity of the relationship with the WAT in terms of the amount of activities logged. **Table 1** provides an overview of the participant sampling. A “0” reflects current non-engagement (due to no longer using the WAT), while a “2” reflects the highest level of engagement.

**Table 1:** Sampling of participants.

Name	Interest in monitoring day-to-day activities	Level of intensity
Joanna	0	0
Christine	0	0
Mary	1	1
Jane	1	1
Sofia	2	2
Catherine	1	2
Paula	2	2
Maria	1	2

There were many other sampling criteria which could have been adopted to select a sample, for example, the reasons for initiating the use of the WAT, types of job/job function (e.g., sedentary versus active), type of WAT (e.g., brand, functional features, position on body), and life stage (e.g., single, couple, family). We chose not to limit the empirical scope of our inquiry beyond engagement level, as we were, primarily, interested in the participants’ day-to-day interactions with the WAT, the collective skills acquisition and the, potential, darker sides of the relationship.

We used two of the authors’ professional networks to identify and obtain access to participants who fulfilled the sampling criteria. This proved a key strength of our study due to the prior familiarity between the interviewer and interviewee, which allowed us to capture personal insight, stories, and experiences, which the participants felt comfortable sharing (Easterby-Smith et al., 2012).

**Table 2** details the participants taking part in the research, including their age group, profession, lifestyle characteristics, activities tracked, and engagement level. To ensure ethical integrity and our participants’ anonymity, each individual was given a pseudonym (Ogden, 2008) and details shared, which could enable others to identify them, were also removed or not used explicitly in the study.

**Table 2:** Participant profiles.

Name	Age	Profession	Lifestyle	Type of tracker	Tracking activities	Engagement level
Joanna	25–30	Business development	Very active and attends the gym several times a week. Highly competitive and driven. Often participated in challenges via the activity tracker. Felt addicted to the tracker. Enjoys team sports. Family orientated.	Fitbit Charge Monitors: Heart rate, Automatic sleep tracking, Steps, Activities, Food and water logging, Distance, Stairs climbed, Caller ID	Used to track: Activities Steps	Used to be a Hyper-engaged user. The WAT needed resetting which has not been done. Has decided not to continue using it.
Christine	25–30	Business development	Very active and attends the gym several times a week. Highly competitive and driven. Often participated in challenges via the activity tracker. Was motivated to challenge others. Felt addicted to the tracker.	Fitbit Charge Monitors: Heart rate, Automatic sleep tracking, Steps, Activities, Food and water logging, Distance, Stairs climbed, Caller ID	Used to track: Steps Sleep Activities	Used to be a Hyper-engaged user. Currently, the WAT is broken and not in use.
Mary	30–35	Human Resources	Very active and attends the gym several times a week. Wants to stay in control and has reduced the number of activities tracked. Wants to be healthy rather than calorie-focused. Does not use the activity tracker app. Accesses data via the tracker itself.	Fitbit Alta Monitors: Automatic sleep tracking, Steps, Smart tracking of activities, Food and water logging, Distance, Move reminders	Steps	Engaged
Jane	55–60	Operations management	Recovering from surgery. Important to do a good number of steps each day. Walking and jazz dance help her to recover and move away from being incapacitated post-surgery.	Fitbit Flex Monitors: Manual sleep tracking, Steps, Activities, Food and water logging, Distance	Steps Water intake Activities	Engaged
Sofia	35–40	Training and development	Highly competitive and enjoys the outdoors. Equestrian at a competitive level. Attends the gym regularly. Walks the dog. More focus on physical activity than gym-based exercise. Always exceeding the targets set.	Jawbone UP Monitors: Heart rate, Automatic sleep tracking, Steps, Activities, Calories, Water intake, Distance	Activities Sleep Steps	Hyper-engaged
Catherine	50–55	Operations management	Very active. Family focused and community orientated. Attends the gym regularly. Is highly aware and interested in fitness and health.	Fitbit Charge Monitors: Heart rate, Automatic sleep tracking, Steps, Activities, Calories, Water intake, Distance, Stairs climbed, Caller ID	Heart rate Steps Sleep Competes against others in work	Engaged
Paula	35–40	Business development	Very active. Recently had knee surgery. Attends team sport. Attends the gym regularly and enjoys weight training. Partner is a personal trainer. Evaluates performance on a weekly basis.	Fitbit Charge Monitors: Heart rate, Automatic sleep tracking, Steps, Activities, Calories, Water intake, Distance, Stairs climbed, Caller ID	Training purposes, specific exercises rather than daily use, heart rate for intensity training Monitors almost everything	Hyper-engaged
Maria	30–35	Program coordinator	Active lifestyle. Often busy on the weekend. Important to always wear the tracker. Highly reliant on tracking sleep. Needs the data to verify daily activity.	Fitbit Charge Monitors: Heart rate, Automatic sleep tracking, Steps, Activities, Calories, Water intake, Distance, Stairs climbed, Caller ID	Sleep Heart rate Steps Activities	Engaged

### Data Collection and Analysis

Participant interviews took place over a period of four months. The interviews were guided by a semi-structured checklist of areas, which were informed by our central research questions. We themed the questions into six categories: About the research participant, About the WAT, Usage, Behavior, Relationships, and Drawbacks/Downsides. Hence, we were interested, not only in the positive aspects of participants’ interactions with the WAT, but also those that were perceived to be of a more negative or challenging nature. Specific questions directed at all participants included whether the WAT had made them feel guilty, underperforming and regretful of looking at the analytics.

The semi-structured interview template was used to ensure that similar areas were explored for each participant for comparability and depth of insight (Irvine et al., 2013). It was important to create a dialog and conversation with each participant, enabling them to speak openly about the experiences that reflect their tracker relationship. Therefore, it was important to create a good rapport with each participant by showing interest in their stories, using listening techniques and asking for clarifications and examples at appropriate times during the interviews (King and Horrocks, 2010). To encourage this conversational and open interview format, we, at times, allowed for participants to influence the direction of the interviews (Stern et al., 1998) and describe situations, experiences, and feelings which came to mind (Thompson et al., 1989). In this way, not all interviews were conducted using the same ordering of questions from the interview template, although all areas were covered within each interview.

The interviews were audio recorded and thereafter transcribed verbatim. Each interview lasted between 45 and 60 min. We conducted a thematic analysis through an inductive process (Corbin and Strauss, 2008). The first stage of the data analysis involved a broad coding of themes that was not confined to specific assumptions or directions to allow for a multitude of interpretations and themes. This was a process initiated by the author unfamiliar with the research participants, who shared the initial themes with the research team. Thereafter the other authors read the transcripts, took notes, and identified initial themes. This was followed by several collaborative research meetings where each member of the research team presented their findings and rationales. From this iterative and collaborative process (Spiggle, 1994), the core themes were refined and agreed on. At this stage, we were highly alert to the themes’ relevance to the central research questions. We focused on those themes that best reflect our participants’ experiences of their interactions with and usage of the WAT and its cognitive resources and how agency becomes distributed across the participants and the WATs.

The validity of the research process can be assessed by the ability of the research team to capture the experiences, actions, emotive responses, and collective skills of the interview participants (Easterby-Smith et al., 2012) and the extent to which the research and analysis methods were effective in addressing the central research questions. The sampling of individuals with varying WAT engagement levels exposed us to a greater breadth of insight, while still focusing on the same core research questions. As all members of the research team were actively involved in the coding, analysis, and theming of the data along with several collaborative research meetings, this enhanced the credibility and validity of the research findings, while also minimizing bias (Denzin, 1989). The main limitation of this approach maybe the reliance on participant self-reporting. In the interviews, participants were required to share their experiences, feelings, and behaviors related to their interactions with the WAT, rather than, for example, presenting the actual WAT engagement reports. It was important for this study to go beyond and behind performance dashboards to explore and understand participants’ subjective experiences and feelings about their interactions with the WAT.

From the iterative and inductive process and guided by our research questions, we identified three themes. With the presentation of these themes, we illuminate how our research participants gain new skills and capacities, ways they become empowered by the WAT and also the darker sides of becoming a human-WAT hybrid.

## Results

This empirical investigation has multiple inter-linked purposes. First, we wanted to explore how our participants and the WATs interacted with a particular focus on the role of the WATs in helping the participants to undertake tasks, solve problems, and gain insight. Second, we focused on the distribution of agency within the human-WAT hybrids to illuminate how the varied expressions of agency affect the human-WAT coupling and thereby also the degree of extended cognition. We relied on the human participants’ experiences of these events, situations, and daily practices.

### Collective and Extended Skills

From the participants’ accounts of their experiences, the WATs, as external resources, have played an important educational role in their lives. They have contributed with new insight about their activity levels, calorie burn/in-take, sleep patterns, and other body metrics. The main interfaces for this sharing of knowledge were the WATs and the WATs’ mobile and desktop software applications. The performance visualizations acted as a gateway to systemized and categorized records of performance that were automatically logged by the WATs (e.g., sleep data) and some which were manually kept updated by participants (e.g., food/calorie/water intake). Initially, participants had found the WATs’ abilities to collate and visually represent the biometric data intriguing and exciting, as they felt they were given access to an “X-ray” of themselves. Catherine, who had been using her WAT for a few months, explained:

I think it is quite interesting. I watch the activity tracker all the time. How many steps I have taken during the day. I think it gets you to be more active. If I have been racing around, I like to also keep an eye on my heart rate too, just keep an eye on it. And the steps, I am also looking at the steps. I try to do at least 15,000 a day (Catherine).

This was a typical reaction among the participants, who believed it was helpful to use the WATs to keep taps on themselves and track their performance throughout the day. As the WATs were set to track the participants, they were able to provide real-time feedback that gave participants instant insight into how their body was reacting to certain activities (e.g., by tracking heart rate) and their progress toward meeting set goals (e.g., by counting steps). Several participants believed that using the WAT to capture, store, and visually present the various biometric data had affected how they went about making certain decisions related to their health management, including eating habits, sleep patterns, and activity levels.

The automation of biometric data collection was mentioned as the primary role and responsibility of the WATs. Participants expected the WATs to have these abilities in order to help them fill in the “blank spots” of knowledge related to their health management. It transpired that all participants had the perception that health-related decisions that were informed by data were better decisions than those simply informed by their own opinions, feelings, and experiences. This belief was a major reason for using and interacting with the WATs. It was clearly the participants’ view that the WATs contributed with a new set of competencies, which assisted them to better understand and assess calories in food items, create and manage sleep routines, and estimate step counts.

The WATs not only contributed to skills related to capturing, storing, and visualizing performance data, but were also expected to provide certainty and reassurance. For Sofia, the WAT was used specifically as an external resource to evidence to herself and others that she not only meets, but exceeds her daily activity targets and therefore has the right to feel tired in the evening. The WAT provided the data and visualizations, which complemented her own internal resources (e.g., ability to remember and explain to others about her daily routines) to feel reassured and to reassure others of her high levels of activity. The process of creating evidence and reassurance was further enabled as the WAT acted as a diary, containing all past performances and goals exceeded. This is data and insight that would have been challenging for Sofia to memorize accurately without the support of some sort of cognitive scaffolding.

I keep getting grief because people say I am tired. But when you walk the dogs before work and you look after two horses after work, generally on a good day, I am not normally home until half 8, 9 o’clock. I was tired. So, it is quite interesting to know how much I am doing each day. I love the sleep data! Telling me how much sleep I have had. I love what it tells me! To have an idea about how much I am doing each day. I normally exceed my targets by 140–150%. So, it is also quite interesting to know that when you say you have had a busy day. I love data like that (Sofia).

Participants tended to trust the data that the WATs collected and presented to them. This trust was an important aspect of the interaction between the participants and their WATs and affected their willingness to respond to and interact with the WATs. By not questioning the data, the WATs were relied on for their information, analytics, and input as external resources and seen as important components toward achieving a higher level of daily activity.

It was evident that the WATs were active in nudging and prompting participants to either adopt or avoid a particular behavior or decision. For some participants, the notifications and performance updates provided by the WATs caused them to make time for a walk during their lunch break, review current decisions on types and amounts of food intake, and systemize movement throughout the day by using alerts. Mary, for example, enabled her WAT to nudge her once an hour to get up and take 250 steps. She felt too sedentary in her job and needed the WAT to remind her to be systematically active. Similarly, Jane had become more conscious of her activity levels after she started using the WAT. She used the WAT to gain a status report of her actively levels at lunch time and if the performance was low, she would make purposive efforts in the afternoon to walk and meet her step targets. In this way, the participants’ ways of thinking and evaluating different actions and options related to their health and well-being, were affected by the WATs ability to track, monitor, store, and present their performance data in real-time.

The participants had acquired their WAT for different purposes. Some sought its support as encouragement to achieve a heightened level of exercise, some looked to the WAT to simply document an already active lifestyle and others wanted the WAT specifically to guide them toward a weight loss. Some wore it day and night to track many different activities, while others wore it mainly at the gym or when completing specific types of exercise (e.g., running). Therefore, some participants set their WAT to track and monitor many different kinds of activities, while others were more selective of when they wanted the WAT to monitor and track their performance.

Despite the different reasons for having acquired the WAT, all participants looked to the WAT as an external source of certainty. The data and visualizations of performance complemented participants’ internal knowledge about healthy eating habits and exercise. It acted to remove some uncertainty by over-ruling participants’ own subjective gut feelings and estimates and provided a perception of an objective truth. The participants’ trust in the WATs’ data capture and visualizations was heightened by their own inability to gather and process this kind of information with a similar degree of accuracy and speed. For Maria, she used the WAT’s sleep tracking feature to monitor the quality of her sleep. She trusted the WAT’s ability to provide a truthful representation of whether she had had a good or a poor night’s sleep and did not question it.

I track sleep because then I can justify why I am tired. I can see that I have had a bad night’s sleep. I didn’t buy the tracker so that I could track my sleep. It is more of a side benefit. But I do like to track sleep to feel justified why I am tired. I feel better when the data tells me that I have had a bad night’s sleep. I don’t like it when I feel like I have had a very bad night’s sleep and my tracker tells me that I have had a good night’s sleep. That annoys me! Then I don’t have an excuse to feel tired. And I believe it. I trust it. So, I have no excuse to feel tired. I just have to get on with it. I know it’s odd! (Maria)

In addition, the overarching purpose for acquiring a WAT was to complement participants’ own abilities to estimate and assess their level of activity, calorie intake, and sleep patterns. They acknowledged that these assessments were often inaccurate, faulty, or simply not possible to undertake and keep track of. This expertise was instead expected of the WATs. For some participants, the WATs helped them to establish new activity routines and give greater insight into calorie consumption, step counts of certain routes and how to achieve better sleep pattern results.

### Human Empowerment Through Technology Extension

Participants expressed how the data and visualizations generated by the WATs made them feel empowered. It was seen as a source of simplification of choices and decisions due to the transparency of performance and progress the WATs provided as and when requested by the participants. The WATs and the related mobile phone application kept participants updated on progress throughout the day and this was experienced to increase their confidence in decision-making.

For Sofia, who already had an active lifestyle before she started using the WAT, the role of the WAT was not to encourage her to be more active; rather the opposite. The role of the WAT was to help her manage her need to live up to societal expectations of how much one ought to weigh, exercise, eat, and so on. The analyses created by the WAT became a “pressure releaser” by alerting her of when she had met the set targets and providing the quantitative evidence.

It tells me “It’s OK, you’re doing enough.” You know you read so much, watch so much about how much exercise you are meant to be doing, what the average is, from a fitness perspective, from a weight perspective and that kind of stuff. And I find myself thinking, I don’t have much more time in a day. Do I go to the gym? No, I don’t have time in a day, unless I maybe take 20 min at lunch to do that. So it is making me go “You do enough Sof.” Instead of telling me that I should be doing more, mine is about taking pressure off, instead of telling me that I need to go for a run (Sofia).

This example demonstrates how the WATs can reduce pressure and stress that participants put on themselves to be active. In fact, it can help participants to make decisions *not* to exercise by confirming that their level of activity is already high and they are meeting the set goals. Here, the WATs contribute with objectivity and analytical evidence to support participants’ otherwise subjective assessments.

Participants tended to either become more reliant on the WAT in order to make health, fitness, and food related decisions, or grow in confidence to make their own decisions with little input or guidance from the WAT. The more dependent participants needed the data to ascertain their own performance and used it as encouragement to continue. Other participants reported seeing their confidence grow as a result of learning from the tracker and acquiring their own, internalized capacity to manage their health and well-being. This indicates the presence of a knowledge transfer, where some participants learn from the WATs’ ability to measure, monitor and track. Participants had gained new knowledge of the length of a particular walking route, the speed at which they walked a mile, the calorie amounts of different foods, and the calories burnt from different types of exercise. This learnt knowledge had built confidence in them to make more independent decisions related to measuring, monitoring, and evaluating their activity levels, eating habits, and sleep. These were abilities that previously were possessed mainly by the WATs. Joanna explained how she is no longer using her WAT, but that it has helped her to establish an active routine, which she has been able to continue with despite not using the WAT.

Beyond providing the performance data, participants particularly enjoyed receiving the WATs’ buzzing vibrations when a goal had been achieved. This provided a tactile interaction between the WATs and the participants that was effective in eliciting a positive emotion in participants, who felt proud and happy to have reached their goals. These vibrations created a physical connection between the WATs and the participants who were able to *feel* the WATs and were alerted to their communication. When the WATs were “silent” (e.g., not vibrating or making a sound), they also became invisible as participants would forget that they were on their wrists. They, however, remained accessible to give updates on performance. Most often, participants would turn to the WATs’ mobile phone applications to gain more in-depth performance updates, whereas the WATs on their wrists provided a quick snapshot of progress. The buzzing vibrations and other indicators of reached goals (e.g., flashing lights, sounds) were ways that the WATs directly interacted with the participants and influenced their actions, behaviors, and decisions in real-time. Participants anticipated these interactions with the WATs and were open to be influenced; whether that was to be reassured (e.g., performance is on track) or encouraged (e.g., to heighten activity levels). They trusted the guidance provided by the WATs and used it in real-time to make decisions.

Some participants even wanted the WAT to take on a more proactive and influential role. Paula explained:

I probably want my tracker to be a scary, sort of, army person. In my ideal world, it would be someone who would be like “Come on, get your act together, let’s get to the gym.” That is the kind of motivation that I like and the kind of motivation that I need as well. I like to be told what to do and “Come on, try a bit harder” (Paula).

The WATs not only used vibrations and sounds to affect participants’ decision-making, but also used the color green to induce behavior change. The green color was used in the WATs’ mobile applications when goals were met. Participants explained how seeing this color, and knowing this was a sign of success, prompted them to feel happy, self-fulfilled, and positive. The green color signaled goal completion and became synonymous with accomplishment and encouragement.

It is a very clever dashboard in the sense that if you have achieved your goals it is in green. I don’t know who has chosen green, but green does make you feel happy, it’s like “Yeah go, you have achieved what you needed!.” If you have only achieved 75% of your target, then it is amber orange. So, it is like a traffic system almost (Paula).

The WATs also used other ways to communicate with and affect participants such as smileys, badges, and trophies, which they received from completing their goals and taking part in competitions.

The WATs also contributed with more socially enabled capacities to encourage participants to be active. Such capacities included step competitions, which allowed participants to compete against other WAT users. The competitions were mainly daily and weekly step challenges and were effective, for some, in increasing activity levels. Participants often competed against colleagues at work and were keen to keep an eye on everyone’s progress. However, not everyone was interested in participating in these challenges. For Joanna and Christine, the competitions had initially provided much fun and excitement, but turned out to be a short-term fad, which did not sustain their interest.

Some WATs were perceived as friendly supporters that mainly offered encouragement, advice, guidance, and data-driven insight. However, for Mary, this was her second WAT. The interactions with her first WAT had been strained, not because there was anything technically wrong with the WAT; it did what it was supposed to do, however, that was exactly the problem. She had felt controlled by the WAT and described it as a “relentless task master.” Comparatively, the new WAT was more like an “ally that cheered her on.” The interactions with the new WAT reflected a different dynamic. She limited the WAT’s influence by carefully managing how she interacted with it and how deeply embedded it was in her daily life. She did this by setting more realistic goals, reducing the number of activities it could track and she requested more infrequent updates from the WAT’s mobile phone application. Consequently, she became less obsessed with knowing her weight and less critical of her physical appearance. She started to appreciate her interactions with the WAT and found it helpful when it vibrated and nudged her to be active because she experienced a greater extent of control. This illuminates that within the human-WAT cognitive system, there is an on-going negotiation over influence, which affects decision-making, control, and competencies.

In this theme, it has been evidenced how participants used the WATs and performance data to gain insight, which, for many, was experienced as a form of self-empowerment and extended ability to make better health-related decisions. However, there are also darker sides of relying on WATs and their resources. In the following section, we present further evidence of the complexities of these dynamic human-tech interactions.

### Darker Sides of Human-WAT Hybridity

We observed that the hybridity with the on-body, always-accessible WAT also led to some negative experiences for the participants. These experiences reflect the WATs’ non-neutrality as well as some of the unintended consequences of an extended mind.

The cognitive abilities that the WATs contributed with (e.g., learning about food calories, calorie burn/in-take, real-time activity levels) made some participants feel insufficient, poor performing, and negative about themselves. Hence, what had started as an exciting experience, turned, for some, into a source of self-loathing and disappointment. Mary explained how she had taken a break from her first WAT because it was constantly reminding her of how she was *not* meeting her targets, which made her feel guilty about her inability to change her behaviors:

I didn’t feel that it [the WAT] was actually working for me and my routine. I think it just kept telling me that I was gaining weight and I got angry and I stopped using it. I think it was the realization of having all that data, it was actually making me realize how unhealthy I was at the time (Mary).

Some participants reported feeling exposed and confronted with what they perceived to be bad habits (e.g., over-eating and a lack of exercise). This led to some emotional distress. By extending monitoring and measuring capabilities to the WATs, it surfaced behavioral traits and habits, which did not match up with some participants’ ideal perception of self. The WATs provided quantifiable data, which previously had been ignored, suppressed or simply unknown to the participants.

As the participants expressed a high level of trust in the biometric data, it led to a sense of *bodily disconnect* for some. This disconnect was expressed as a form of alienation between the participant and her own body, fueled by an increased uncertainty about how to best manage and build a strong and healthy body. One participant explained this by saying that she had stopped listening to her internal body and had become reliant on what the WAT told her. The perceived superiority of the WAT to provide a more truthful and accurate assessment affected especially those participants who had become reliant on the WAT’s abilities to capture, store, and analyze their data. Some participants were reliant on the data to confirm that they had indeed completed the particular activity. Hence, when the WAT was physically absent, which also led to an absence of the data, they experienced a reduction in the ability to monitor, measure, and assess their activity levels. In this way, participants did not feel capable of completing the tasks that the WATs could undertake.

If I forget to charge it or forget to put it on then I get very annoyed. Because then I have done steps but there is no data to prove that. I don’t like that. It is a very bad habit. One day I did a lot of activities and I had forgotten to put it on and I felt very disappointed, even though it is just me who looks at the data. I want to have the data. If you have done the steps and you haven’t recorded it, have you really done it? (Maria).

Some participants had developed an intensive dependency relationship with the data, feeling obsessed with checking it. This included tracking progress, analyzing performance, and responding to the data. It offered the capability not only to track performance in real-time, but also to store this data for later comparisons, evaluation, and analysis. This meant that participants’ interactions with the WATs were not just real-time, but also involved understanding longer term performance trends. Joanna reflected on how the cognitive capabilities provided by the WAT had led to a sense of obsession:

It was obsessive. It would be all you thought about. You would be constantly checking your steps. How many have I done? You’d walk to the toilet and then check how many steps that was. Always refreshing the app to see if anyone else had done more steps. I’d be like “Oh no she is ahead of me I need to go for a walk.” It definitely disturbed me during the day. I’d have my phone on my desk, so I’d be like “Let’s have a little look” when its after lunch, because people would have gone to the gym and I’d be like “Oh I need to go for a walk.” So yeah, it probably didn’t help productivity in my work life (Joanna).

The extended capabilities of the WATs were not always a positive influence on participants, but led to feelings of stress, self-blame, and the need to improve the performance data even if it was inconvenient or unwanted by the participant. Jane explained how the presence of the data made her determined to meet her targets and when she did not achieve these, she felt angry with herself. She put additional pressure on herself to catch up with the “lost steps” the following day.

I feel cross with myself. All I had to do was to go around the block to meet my target. I should have gone around and done that. I was a bit cross with myself. I should have done that and today I’ll try and do 12,000 steps to make up for yesterday (Jane).

The need to reach targets was intensified if targets were part of a competition with other WAT users. Christine and Paula explained how the new capability of tracking and quantifying their exercise and being part of competitions had become a dominant influence on their lives:

It is ridiculously addictive, actually got a bit stupid because it started to interfere with my life and if I hadn’t reached a certain amount it would be like 10 o’clock in the evening and I would be like, “I’m going for a run.” I’m going out just so that I can get my steps up, just to beat the people that I was in a competition with. Your life had become a constant challenge (Christine).

It is addictive. So, at the very beginning, reaching the goal of the steps was quite important. If someone invites you into a challenge then it gets quite competitive and you do try and beat the other people, especially when it is a narrow margin (Paula).

Some participants took action to change the capabilities of the WAT. They did not stop using the WAT as an external feature, but they purposively reduced the WAT’s ability to influence them. Participants explained a number of moves taken to change the outcomes of the WAT’s biometric data processing. Some participants inputted a lower food calorie amount for the items eaten to keep their daily calorie intake below the maximum target set on the WAT. Some kept the WAT’s step count target purposely low to ensure that they would meet, or even exceed, the target. This would then trigger the WAT to congratulate them on their achievements, consequently inducing positive emotions. Others were strategic about when to request the WAT to sync their performance data from the WAT wristband to the mobile application. In particular, when competing in challenges/competitions, participants requested this sync of data only on the final day of the challenge/competition so not to reveal their progress to the others in the competition. Others were selective about the types of activities they requested the WAT to track, de-selecting some low-performing categories (e.g., sleep and high-intensity activities).

The influence of using the WAT as external scaffolding also became apparent in how the WAT was able to make visible specific performance targets. The mere presence of these targets created the expectation that these needed to be met. When they were not met, it often elicited feelings of frustration and disappointment for participants. Feeling that these performance targets needed to be met meant that participants, at times, adopted behaviors and actions which they would have rather not. Mary, for example, explained how the ability of her first WAT to monitor and assess her eating and drinking habits made her feel disempowered. As she inputted her food and drinks consumption, her habits became quantified, stored, and visually presented to her in the WAT’s mobile application. These were habits that had not previously been a concern to her, but the trend data presented by the WAT made her feel embarrassed. It came to a point when she could no longer continue with her normal eating and drinking habits without a high amount of self-doubt. Consequently, the WAT became a source of self-loathing.

It came to the point where I was thinking “Do I have those two glasses of red wine or not?” I was too embarrassed, my Fitbit embarrassed me every day (Mary).

In summary, the investigation revealed that participants’ experiences of using WATs to undertake specific monitoring, tracking, and analytical tasks were not without challenges. Darker sides of attaining extended cognitive abilities transpired in the form of self-doubt, bodily alienation, and a mindset fixated on goal/target completion. As a consequence, all participants made moves to limit the WATs’ influence and/or the accuracy of its analytics by adjusting the biometric or food input. This was a way of managing the new situation, where cognitive processes related to health management were supported by the abilities of external devices.

## Discussion

The study demonstrates that our relationship with technology is complex, even when the technology is relatively mundane and simple, such as WATs. In this study, the EMT has been shown to be an effective analytical apparatus to explore how cognition can become extended beyond the human and the impact this has on decision-making, behaviors, and experiences. The EMT is best adopted as a theoretical lens when the distribution of cognition occurs in relation to specific tasks for which the human needs the support and resources of external entities.

This study builds on the EMT by examining the non-neutrality of the WATs and how they acquire agency in particular situations. Agential intra-action supports the view that external entities are not neutral or non-expressive, but can, in their intra-action with others, for example humans, become expressive, shape intent, and action (Barad, 2003). This dimension of human-tech relationships is important alongside the EMT as it helps us to illuminate, not only distributed cognition, but also distributed action and effects. With our empirical investigation, we have contributed with insight on human-tech hybridity, and also demonstrated how the EMT and agential intra-action can be used in conjunction to drive knowledge in empirical studies.

It has become evident that those who use a WAT interact with it with the purpose of generating personal data and insight that can lead to certainty and which would be challenging to attain without the support of a WAT. This is similar to findings in the work of Schroeder et al. (2018) who investigated how people use self-tracking technology to better manage migraines. It was found that many people track symptoms and use app alerts to try to predict when a migraine is likely to come on, hence empowering the person to reduce the factors leading to a migraine or better prepare for it. In this way, the self-tracking technology, much like the WATs, is an external resource that can enhance people’s capabilities to predict and make decisions.

The WATs become influential external resources in an environment where individuals are focused on identifying the best ways to manage their health and well-being and struggle to trust their internal instinct and gut feel to do so. Hence, the WATs are used as scaffolding (Norman, 1993) to reduce perceived uncertainty and gain support in decision-making.

**Table 3** provides an overview of the types of tasks where the WAT offer its resources to complement the human cognitive abilities through enabling extended memory, data capture, and analytical capabilities. The WATs supplement the humans with additional resources, which make the humans better able to judge and take decisions about how to attain a certain calorie intake, maintain a certain intensity of activities (e.g., steps, calorie burn, movement notifications), and review and assess past performance trends. While an individual could capture much of the information related to exercise and food/drinks consumption using a manual logging method, the WATs provide efficiency, automation, objectivity (unless the human manipulates what is logged), consistency, and real-time analytical capabilities based on large and diverse sets of data. Moreover, was the human to undertake these tasks manually, the tools used to capture the information, e.g., notebooks and pens, would also become external scaffolding.

**Table 3:** WAT resources supporting and enabling human cognitive processes.

Task	WAT resources	Human cognitive processes
To capture, store, analyze, systemize and categorize real-time biometric data	The ability to record, store, analyze and visually present performance data. The ability to undertake various analyses of the data based on the human’s request.	The ability to operate the WAT, manage settings and enable/disable the WAT’s biometric data collection abilities. The cognitive ability to set up a sync between the WAT, the WAT’s mobile phone app and the WAT’s desktop interface. The cognitive ability to set health and activity goals.
To ascertain times when an increase in movement/exercise is recommended	The ability to manage large biometric datasets, undertake pattern recognition analysis, compare performance with set goals, and provide notifications/nudges.	The ability to judge whether to respond to the notification/nudge, which may depend on other situational factors.
To monitor and track calorie intake	The ability to store, categorize and visually present consumed calories over time and according to food/drinks categories.	The ability to identify the best match between a selected food/drinks item and food/drinks items available on the WAT’s mobile app or desktop interface.
To gain insight into sleep patterns and rhythms	The ability to automatically detect when sleep is initiated (or respond to sleep mode being activated) and record hours slept, sleep patterns and present a daily analysis of sleep quality.	The ability to manage the sleep mode setting.
To monitor heart rate in real-time	The ability to monitor and record heart rate in real-time, quantify heart rate performance, and provide notifications/nudges when deemed above an advisable level.	The ability to judge whether a change in behavior is required to either increase or reduce the heart rate.
To gain certainty about the activities required to attain a healthy and active lifestyle	The ability to generate real-time and quantitative information that reflects in-the-moment performance as well as analyses that are based on longitudinal performances.	The ability to process the information and analyses produced to complement own information processing capacity.

Our study found that initially the WATs are exciting external entities that enable access to biometric information, considered to give new and enhanced decision-making abilities. It is an example of a bio-external device (Clark, 2015) that is flexible and individualized in the way it can share its resources (e.g., data trends, visualizations, notifications, goals) with the individual who draws on its resources and capabilities. In this way, the interactions between the individual and the WAT are dynamic, as the more the person uses it and also manually logs activity, the more data the WAT has to capture, store, analyze, and make available to the person [see also Rapp and Tirassa’s (2017) work on a new theory of the self and related guidelines for the design of personal informatics technology].

In most situations, the analyzed and visualized outputs are considered to be trustworthy, which is based on the individuals’ implicit trust in the data (Arango-Muñoz, 2013). The individuals do not question what the WATs tell them. Although in some cases, an individual may not like the outputs that the WAT presents them with, for example, a display of poor sleep patterns, and chooses for the WAT to stop monitoring this activity. This, however, is typically not because the data is mistrusted, but often because it is disliked. In addition to trustworthy, the data outputs are also considered to be reliable and, due to the WATs’ on-body position, accessible at almost all times. These factors have an impact on the strength of the coupling (Heersmink, 2017) between the person and the WAT.

Coupled systems of heterogeneous entities can create new cognitive systems, where each entity takes on an active role (Clark and Chalmers, 1998). In this study, it is evident that both the WATs and the individuals co-constitute new hybrids that have emerged as a consequence of the human-WAT intra-actions (Barad, 2003). The WATs affect, and for some transform, the experience of being-in-the-world, as they provide a new layer of quantified “life data.” At the same time, the individuals also contribute to the coupling by giving the WATs access to trackable and quantifiable behaviors and allowing the devices to be present throughout the day and, for some, the night. However, Rapp and Tirabeni (2018) point to a potential loss of agency and control a person may feel over own body when engaging in self-tracking activities. In their study on mechanisms of externalization of the body among amateur and elite athletes, it appears that elite athletes are better at regulating their interactions with the tracker and know when to trust their subjective sensations, whereas amateur athletes are more reliant on the data to assess their performance. Hence, it seems that the human-WAT relationships may also be affected by the human’s existing “practice in relation to her body” (Rapp and Tirabeni, 2018, p. 14).

In this study, it was observed that the WATs have attained an ineliminable role in most human-WAT hybrid relationships (Clark and Chalmers, 1998). This is seen in how the collective competences acquired by the human-WAT hybrids are affected when the WATs are absent. This absence leads to an inability to accurately estimate the number of steps taken, calories burnt, and other data, which the WATs normally collate. This loss in ability is not something the individual believes he or she can restore through their own cognitive abilities. The absence-induced reduction in cognitive information gathering and processing further highlights what it is that the WATs impact and contribute with (Tripathi, 2010); namely, the ability to record, store, analyze, and visually present, at the individual’s request, insight into the individual’s health and activity performance and progress. Not all people, however, feel unable to make estimates about their activities when the WAT is absent. Some have internalized new behaviors as a consequence of interacting with the WAT and have, as a result, heightened their confidence in making health-related decisions without the input from the WAT. Therefore, when the WAT is absent, they become less affected and are able to make unassisted estimates related to calorie intake, distance covered, and the quality of their sleep.

From the participants’ accounts, the access to activity and performance data helped to create a sense of heightened control and reduced fear of making poor decisions (Schulz, 2011) related to their health. Schüll (2016) emphasizes that self-tracking devices are both sources of responsibility and delegation. People who self-track wish to make informed decisions and take responsibility for their actions and behaviors, but at the same time, they also delegate part of the responsibility for this to an external device. Specifically, what is delegated to the external technology is the responsibility to “calculate and act upon itself.” This is seen in how the WATs are given the responsibility for calculations and assisting the individual’s decisions through nudges and notifications. However, the darker sides of the human-WAT hybrid also reveal that relying on the resources of a WAT is not uncomplicated.

Drawing on the views of Ihde (1990); Pickering (2013) and Heersmink (2017) in terms of the non-neutrality of technology and the belief that technology, like humans, can also express agency, we contribute with the concept of the *agency pendulum*. The agency pendulum draws inspiration from Barad’s (2003) rendition of agency; that it is enactments which emerge from intra-actions between all sorts of entities including those that are not human. Hence, agency is not a constant and it is not an attribute assigned to an entity (Ewalt, 2016). Instead, it is played out, expressed, and seen through effects and collective capabilities (Pickering, 1995).

The agency pendulum swings between the human and the WAT, which means that, at times and in specific situations, the human is enabled to affect and create change; in other situations, it is the WAT that influences and impacts decisions and behaviors (**Table 4**). The agency pendulum does not reflect a symmetrical division of agency. Its movements are individualized to the specific human-WAT hybrid and are affected by, for example, how much the human cares about, listens to, and becomes affected by the expressions of the WAT as well as the expressive abilities of the WAT. The agency pendulum acts as a metaphor for distributed agency and attempts to highlight the non-neutrality of the WATs as they intra-act with the humans.

**Table 4:** Agentic enactments.

**Human**
• Change the settings to increase or decrease the number and types of metrics being tracked
• Prevent the WAT from synching with the mobile phone app or WAT’s desktop interface
• Alter the WAT input data to generate particular results from the WAT’s analysis
**WAT**
• Send real-time notifications and nudges that lead to behavior change or affect decision-making
• Share health data that alter attitudes toward food and drink consumption
• Gain trust as an objective and truthful reflection of health performance

The human enacts agency in situations when the individual limits or extends the WAT’s presence and influence by increasing or decreasing the collection of particular biometric data. Nafus and Sherman (2014) describe these kinds of practices as forms of “soft resistance” to the automatic collection of personal informatics. The soft resistance becomes visible when people purposely choose when automatic data collection is a helpful external exercise and when they would rather use internalized decision-making criteria. In our study, human agency is also expressed when the WAT is prevented from synching the captured data with the mobile phone app or desktop interface. This prevents the WAT from undertaking the analysis and visualization of the data, which is a central task. The human is also seen to express agency when the data inputted is altered to achieve certain outputs. This could be food calorie data that is lowered or a step count target that is set purposely low. In these situations, the agency pendulum swings toward the human, as the human carries out acts that affect him/herself, the intra-action with the WAT and the influence that the WAT can have. These acts challenge the “reflexive monitoring self,” which is often described as a “rational, motivated, and data-centric” individual (Lupton, 2016, p. 115). Rather, at times, the human acts to reduce or alter the pattern of tracking and consequent self-surveillance.

When the agency pendulum swings toward the WAT, it is often when the WAT makes expressions, which end up affecting the human. These acts are outcomes of the WAT’s design, but nonetheless, when they are expressed, they have an influence on human behavior and decision-making. When the WAT sends notifications and nudges the individual to adopt or avoid a particular behavior or make a particular decision, then that is an assertion of the WAT’s agency. At times, this leads participants to feel a loss of control over own actions, a wish to reclaim control and an experience that the decisions they are making are not truly their own. Similarly, when the WAT presents food and drinks consumption trend analytics, then this is a situation that can impact the individual’s decision-making or feeling about self. The WAT also enacts agency when it acquires a position of trust, i.e., the human considers it trustworthy and is willing to trust the information it communicates based on its information processing and analytics. The WAT is provided the autonomy to conduct these calculations and visualizations and communicate them to the human, taking on the position of an autonomous system (Ohlin and Olsson, 2015).

The agency pendulum acts as a helpful metaphor when exploring this intersection of distributed cognition and distributed agency. It enables an illumination of the impacts and influences of the external features beyond their roles as offering cognitive scaffolding. It can add a further dimension of insight to understand the dynamics of human-tech hybridity by including a focus on distributed action and impact. Considering the EMT and agential intra-action as part of a connected exploration, enables an investigation that examines both how cognition is extended to other entities to solve tasks and also how these external entities, in their intra-actions with the human, come to enact agency, i.e., become expressive, shape intent, and action. Hence, we put forward the notion that distributed cognition and distributed agency are not capacities that are mutually exclusive, but are in fact closely tied together.

## Conclusion

The EMT and the concept of agential intra-action can be used as a combined theoretical apparatus to explore the distribution of cognition from the human to other external entities and the dynamism of agentic enactments, as both the human and the external entities attain the ability to influence, impact, and create change. The theoretical lenses provide specific concepts that can be applied to surface how abilities, competences and capacities can become co-constituted in human-tech hybrid relationships and how these affect the ability to make decisions and solve problems. The research identified specific ways in which the WATs support the humans’ cognitive abilities by contributing with an extended memory, data capture and analysis capabilities.

The research also highlighted that, while technologies, such as WATs, can contribute with new abilities and insight, there are unintended implications of this engagement between the human and the WAT. Attaining an extended mind and interacting with an external entity that has the ability to influence, guide, and illuminate health-related behaviors through constant monitoring and tracking, can lead to some experiences of stress, disappointment, and self-blame. Hence, as entities and new technologies are developed to support human cognition, it is important to also consider the side effects and non-neutrality of the technology and how that impacts the human experience.

To further capture and conceptualize the dynamism of distributed agency, we presented the concept of the agency pendulum. The agency pendulum swings between the human and the external entity, which means that, at times and in specific situations, the human is enabled to affect and create change; in other situations, it is the external entity that influences and impacts decisions and behaviors.

We posit that it is particular useful to consider distributed agency as an additional layer of theoretical exploration when considering the EMT in order to also capture the non-neutrality of the external entities that support human cognitive abilities.

## Ethics Statement

This study was carried out in accordance with the recommendations of the Ethics Committee at Ashridge Executive Education at Hult International Business School with written informed consent in accordance with the Declaration of Helsinki.

## Author Contributions

This study is part of on-going research into wearable and other digital technologies by RD and MC. RD and MC developed the theoretical framing, undertook the empirical data collection, and drove the analysis and presentation of themes and contributions. NP took part in the data analysis and initial identification of themes.

## Conflict of Interest Statement

The authors declare that the research was conducted in the absence of any commercial or financial relationships that could be construed as a potential conflict of interest.
